# The Potential of Natural Compounds in UV Protection Products

**DOI:** 10.3390/molecules29225409

**Published:** 2024-11-16

**Authors:** Jovana Milutinov, Nebojša Pavlović, Dejan Ćirin, Milica Atanacković Krstonošić, Veljko Krstonošić

**Affiliations:** Department of Pharmacy, Faculty of Medicine, University of Novi Sad, Hajduk Veljkova 3, 21000 Novi Sad, Serbia; jovana.milutinov@mf.uns.ac.rs (J.M.); dejan.cirin@mf.uns.ac.rs (D.Ć.); milica.atanackovic-krstonosic@mf.uns.ac.rs (M.A.K.); veljko.krstonosic@mf.uns.ac.rs (V.K.)

**Keywords:** sunscreen, SPF, natural UV filters, flavonoids

## Abstract

Overexposure to ultraviolet radiation mainly leads to skin disorders (erythema, burns, immunosuppression), skin aging, and skin cancer as the most serious side effect. It has been widely accepted that using sunscreen products is an important way to protect against the harmful effects of UV rays. Although commercial sunscreens have constantly changed and improved over time, there are emerging concerns about the safety of conventional, organic, UV filters due to adverse effects on humans (such as photoallergic dermatitis, contact sensitivity, endocrine-disrupting effects, etc.) as well as accumulation in the environment and aquatic organisms. This is why natural compounds are increasingly being investigated and used in cosmetic and pharmaceutical sunscreens. Some of these compounds are widely available, non-toxic, safer for use, and have considerable UV protective properties and less side effects. Plant-based compounds such as flavonoids can absorb UVA and UVB rays and possess antioxidant, anticarcinogenic, and anti-inflammatory effects that contribute to photoprotection. Apart from flavonoids, other natural products such as certain vegetable oils, carotenoids, stilbenes, and ferulic acid also have UV-absorbing properties. Some vitamins might also be beneficial for skin protection due to their antioxidant activity. Therefore, the aim of this research was to gain insight into the potential of natural compounds to replace or reduce the amount of conventional UV filters, based on recent research.

## 1. Introduction

Solar radiation is undoubtedly important for maintaining life on Earth [1]. Solar radiation consists of a broad spectrum of electromagnetic radiation from 100 nm to 1 mm including ultraviolet (UV), visible, and infrared (IR) light. UV radiation is divided into three categories dependent on wavelength: UVC (100–280 nm), UVB (280–320 nm), and UVA (320–400 nm) rays [2]. As the wavelength of UV rays increases, their energy decreases. This means that UV rays with shorter wavelengths, such as UVC rays, are more harmful due to their higher energy [3]. However, the ozone layer absorbs UVC rays and prevents them from reaching the Earth’s surface, which is not the case with UVA and UVB rays [4]. UV radiation has some beneficial effects on humans. It is necessary for the endogenous synthesis of vitamin D, improves cardiovascular health, and possesses antimicrobial activity [3]. On the other hand, UV radiation is one of the stress factors for humans, especially for the skin, so it can lead to skin disorders (erythema, burns, immunosuppression), skin aging, and skin cancer due to overexposure [3,5]. UVA rays can penetrate deeper into the skin than UVB radiation. As a result, UVA rays can reach the dermis and generate reactive oxygen species (ROS) that damage DNA and lead to skin aging [6]. On the other hand, UVB rays directly damage DNA by forming pyrimidine dimer and pyrimidine (6–4) pyrimidone products, which can lead to apoptosis or DNA replication errors. UVB rays may trigger sunburns and erythema, while both UVA and UVB radiation can cause skin cancer [6,7].

Skin cancer is the most serious side effect of excessive sun exposure. Multiple studies have indicated that frequent occurrences of sunburn throughout life are associated with an increased risk of skin cancer [8,9]. There are two types of skin cancers: melanoma skin cancer (MSC) and non-melanoma skin cancers (NMSCs). Skin cancer is one of every three cancers diagnosed worldwide, of which NMSCs are the most common. The highest incidence rates of skin cancers were recorded in New Zealand and Australia, while Europe has the highest incidence and mortality rates of MSC. On the other hand, North America and Asia have the highest incidence and mortality rates of NMSCs [10].

Regular use of sunscreen may reduce the risk of both MSC and NMSCs [11,12]. The use of sunscreen is one of the most important ways to protect against the harmful effects of UV rays, but it is not the only and sufficient. It is also necessary to dress appropriately, wear a wide-brimmed hat and sunglasses, as well as stay in the shade [6].

The first modern sunscreen, known as “Gletscher Crème” was developed and commercialized by the Swiss chemist Franz Greiter in 1946 [13]. Over time, commercial sunscreen products have constantly changed and improved based on advances in scientific knowledge [14]. An ideal sunscreen should effectively protect against both UVA and UVB radiation, be photostable, and be non-toxic for humans, animals, and the environment [15]. In addition, it should form a stable film on the surface of the skin to prevent permeation into deeper skin layers and the bloodstream [16]. The efficacy of a sunscreen is usually measured by the sun protection factor (SPF), which is a measure of how much more UV radiation is required to produce sunburn on protected skin relative to unprotected skin. Namely, SPF corresponds to the number of times the sunscreen increases its capacity to delay the formation of erythema due to sun exposure, compared with unprotected skin [17].

However, despite the benefits of using sunscreens to prevent the harmful effects of UV radiation, the public is concerned about their safety for both humans and the environment. In fact, many studies have confirmed that some UV filters (mainly commercial) have human toxicity, and their residues have been detected in many waters, aquatic organisms, and soil. Based on this, the development of new, safer, and more effective photoprotective agents is being considered [18]. Over time, natural agents have gained considerable attention in cosmetic and pharmaceutical sunscreens [19]. They are usually widely available, non-toxic, safe for use, and offer UV protection comparable to conventional UV filters, as well as antioxidant, anti-inflammatory, and immunomodulatory properties [20]. Therefore, the aim of this research was to gain insight into the potential of natural compounds to replace or reduce the amount of conventional UV filters, based on recent research.

## 2. Types of UV Filters

**Figure 1:**
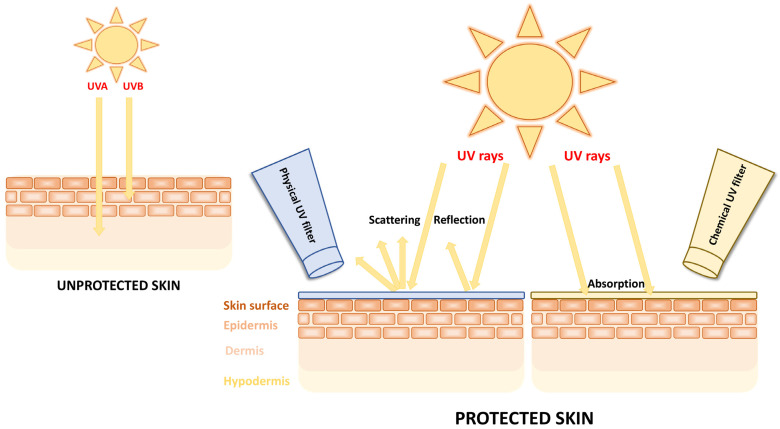
Mechanism of action of physical (inorganic) and chemical (organic) UV filters.

UV filters are generally categorized into physical (inorganic) and chemical (organic) filters according to their physicochemical properties. Physical filters usually reflect and scatter UV radiation, while chemical filters absorb light (Figure 1) [21].

Inorganic UV filters include talc, bentonite, kaolin, and silica, as well as titanium dioxide and zinc oxide as the most commonly used [4]. The inorganic filters are called physical because of their mechanism of action, reflection, and scattering, which are physical phenomena [22]. However, they also can absorb UV radiation to some extent [23]. Only two inorganic filters, titanium dioxide and zinc oxide (also known as mineral filters), have been approved by the Food and Drug Administration (FDA) as safe and effective UV filters [24,25], as well as by the European Union (EU) [26]. The inorganic filters have some advantages over the organic ones such as broad-spectrum UV protection, both UVA and UVB protection and photostability, while some organic UV filters are not photostable and may produce irritating or sensitizing metabolites that can lead to allergic skin reactions and skin irritation [27]. Although they are the preferable option for sun protection for children and people with sensitive skin, they can leave a white pigment on the skin and stain clothes due to their ability to reflect visible light and the large size of the particles. Therefore, inorganic filters, in commercial sunscreens, are micronized and nanosized to achieve an acceptable appearance and enhance the UV protection effect [5,27]. Moreover, these filters are insoluble in water and can break the emulsion, so it is challenging to formulate products based on inorganic UV filters [21].

**Figure 2:**
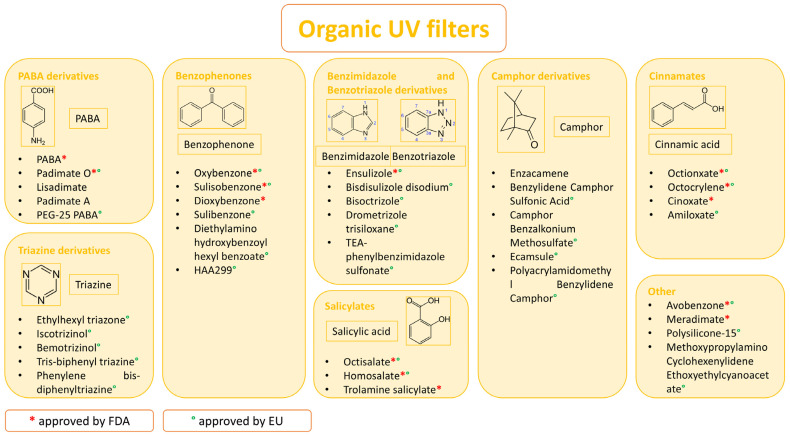
Classifications of organic UV filters.

Different classifications of organic filters are available in the literature, but they are all based on their structure. One of these is the classification (Figure 2) into para-aminobenzoic acid (PABA) derivatives, triazine derivatives, benzophenones, benzimidazole and benzotriazole derivatives, salicylates, camphor derivatives, cinnamates, and “other” [28].

Organic UV filters are also classified into UVA (benzophenones, avobenzone, meradimate, etc.), UVB filters (PABA derivatives, cinnamates, salicylates, octocrylene, etc.), or broad-spectrum filters (ecamsule, drometrizole trisiloxane, bemotrizinol, bisoctrizole) [3]. These filters are more likely to cause adverse skin reactions than inorganic filters [25]. Moreover, studies have shown that combining multiple organic filters provides broad-spectrum UV protection but decreases photostability [5].

The FDA published a final sunscreen monograph in 1999, which included the 16 sunscreen active ingredients that can be used in over-the-counter (OTC) sunscreen products. In the following years, some of these ingredients have been subjected to changes in their regulatory status. In 2019, the FDA issued a proposed rule on sunscreens, which included new information about the safety of certain sunscreen active ingredients, among other things. They proposed that sunscreen active ingredients be categorized into three categories: Category I is generally recognized as safe and effective (GRASE) for use, Category II is not GRASE for use, and Category III requires additional data before a GRASE determination can be made. For ingredients in Category II, the FDA evaluated that the risks associated with the ingredients outweigh their benefits. Table 1 shows compounds that are recognized as active ingredients in sunscreen products for OTC human use by the FDA, as well as their chemical structures and categories.

## 3. Safety of UV Filters

Previous studies revealed that the UV filters present in sunscreens have some disadvantages related to their photostability, environmental impact, and human toxicity [18].

The small size and large surface area of inorganic UV filter nanoparticles raise concerns about potential absorption through the skin. Nanoparticle exposure to UV radiation can lead to the generation of ROS radicals and possible skin damage [29]. To overcome photoreactivity, nanoparticles of inorganic UV filters are coated with silicon or doped elements (Al_2_O_3_ and Zr) [3]. Osmond-McLeod et al. [30] have shown that the concentration of 68Zn in the organism of hairless mice treated with sunscreen containing nanoparticles is higher than in mice treated with sunscreen containing larger particles (both sunscreens were 20% *w*/*w*). However, they pointed out that the hairless mouse model used has much thinner skin than humans and that skin penetration is easier. Fajzulin et al. [31] have reported that nanoparticles of inorganic UV filters can cause damage to the bacterial cell membrane, but cannot penetrate through intact healthy skin. Filipe et al. [32] have examined the penetration of ZnO and TiO_2_ nanoparticles into normal and psoriatic human skin in vivo from three marketed sunscreens. The results indicated that after 2 h of application, the nanoparticles were only detectable in the stratum corneum. Moreover, after 48 h of application, the nanoparticles were not in deeper skin layers, thus it was concluded that nanoparticles cannot reach viable skin tissues. However, various studies published over the decades show that inorganic UV filters are safe and preferable to organic ones [5].

Previous research has pointed out that certain organic UV filters have detrimental impacts on the environment, animals, and human health. Regarding environmental impact (Table 2), studies have found that certain organic UV filters bioaccumulate, particularly in marine organisms, and are detected in water, sediment, and wastewater.

Organic UV filters have low photostability compared with inorganic ones as previously mentioned, therefore leading to the formation of toxic and reactive photodegradation products that potentially cause toxic and allergic reactions in humans. The double bond of the α,β-unsaturated system presents in the chemical structure of some UV filters, such as cinnamate derivates, which can react with the skin proteins and lead to skin sensitization reactions and allergic contact dermatitis [18]. Moreover, it has been noticed that some organic UV filters (BP-3, Enzacamene, OMC), after topical application (three sunscreens at 10% *w*/*w* of each), can reach the systemic circulation and be detected in the urine [50]. Several studies have reported that UV filters have endocrine-disrupting effects, including estrogenic, androgenic, and thyroid activities [51,52,53,54]. Ghazipura et al. stated that BP-3 can be absorbed through topical application, and humans often ingest it through water intake [55]. Therefore, Tang et al. investigated reproductive toxicity in pregnant women prenatally exposed to BP-3. They found that pregnant women with a high urine BP-3 concentration had a reduced duration of pregnancy compared with women with a low urine BP-3 concentration [56]. Philippat et al. noticed an association between maternal urinary BP-3 levels and increases in infant birth weight [57]. Various studies use in vivo animal models to investigate other negative effects of organic UV filters. Balázs et al. [52] found that BP-3 has a cytotoxic effect on zebrafish. They revealed that BP-3 can cause mortality (LC_50_ = 3.8 mg/L), failure to hatch (EC_50_ = 12.39 mg/L), and various malformations in the zebrafish embryos. Mustieles et al. [54] have reported the endocrine-disrupting effect of BP-3 in in vivo rodent models. Chen et al. [58] investigated the adverse effects of BP-3 using an in vivo model of anemonefish and noticed that BP-3 can cause behavioral changes. In addition, it was found that BP-3 [59] and OMC [60] can be neurotoxic to zebrafish.

## 4. Natural Compounds as UV Filters

As consumers are more aware of the harmful effects of certain pharmaceutical and cosmetic products on both human health and the environment, therefore changing their mindsets, they increasingly seek for sustainability through their purchases [61]. Thus, today there is a growing need to find alternatives to the commercial ingredients of formulations in order to satisfy consumer demands. In recent years, researchers have shown an increasing interest in the use of natural ingredients in the cosmetic industry and state that such products are the future of cosmetics, which entails the use of natural UV filters [19]. Natural compounds are typically regarded as non-toxic and non-irritating and show less side effects on the skin than synthetic ones [20]. They have greater tolerability and a negligible impact on the environment [62]. For instance, natural polyphenols, known for their effectiveness against UV-induced oxidative stress, are also present in the diet, so they produce less sensitization effects than chemically produced sunscreens [62].

Table 3 shows the chemical structure of some natural compounds that have UV protective properties and their absorption peaks.

### 4.1. Flavonoids

Flavonoids are the secondary metabolites of plants and are found in many fruits and vegetables. They protect plants from harmful UV radiation by absorbing the sunlight and scavenging UV-generated ROS [62]. The flavonoids have two maximum absorption peaks between 240 and 280 nm and another between 300 and 500 nm [20]. The presence of phenolic hydroxyl groups in the structure of flavonoids enables them to scavenge ROS [63]. In addition, flavonoids exhibit anti-mutagenic [64], anticancer [65], and anti-inflammatory [66] activities.

#### 4.1.1. Quercetin

Quercetin is a natural flavonol belonging to the subclass of flavonoids, usually present in plants in the form of its glycoside, less often as an aglycone. It is found in various edible fruits and vegetables such as apples, black grapes, cherries, berries, onions, kale, and many others [67]. Quercetin and its derivates have a wide range of pharmacological activities including anti-inflammatory, antidiabetic, antimicrobial, antiviral, and anticancer activities [67,68]. Furthermore, quercetin possesses UV-absorbing properties (primarily UVA radiation), thereby preventing the formation of ROS and direct DNA damage, and also possesses antioxidant activity which could contribute to UV protection [69]. Quercetin shows a maximum absorbance in the UVA range at 375 nm, as well as in the UV-C range at 258 nm [70]. Generally, quercetin has the highest antioxidant activity among flavonoids [71] and can scavenge ROS, inhibit lipid peroxidation, and bind transition metal ions to form inert chelate complexes [72,73]. Erden Inal et al. have shown by in vivo test on rats that quercetin reduces the amount of malondialdehyde, which is produced during oxidative stress, by scavenging ROS and breaking chains, whereby it protects against UVA-induced photodamage. Quercetin was administered intraperitoneally in a volume of 1.25 mL and concentrated at 1% (*w*/*v*) [74]. Also, quercetin administered intraperitoneally in rats prevents the decrease in activities of antioxidant enzymes glutathione peroxidase, glutathione reductase, catalase, and superoxide dismutase after UVA irradiation [74,75]. Zhu et al. have demonstrated in vivo that topical application of quercetin prevents UVB-induced skin damage [71].

#### 4.1.2. Rutin

Rutin (quercetin-3-O-rutinoside) is the glycoside form of quercetin, a widespread flavonoid in fruits and plants such as tangerine, lemon, orange, grapefruit, lime, and buckwheat seeds [17]. In the literature, rutin has been shown to increase the SPF value of conventional organic UV filters significantly [76,77,78,79]. Aside from that, like other flavonoids, rutin has antioxidant activity, which was proven by Tomazelli et al. using an in vitro assay [79]. Also, de Oliveira et al. showed that formulations with 0.1% (*w*/*w*) rutin scavenged considerably more free radicals than formulations without it by in vitro assay [76].

Choquenet et al. [80] have examined in vitro the SPF and the UVA protection factor (UVA-PF) values of quercetin and rutin, as well as the occurrence of synergism and additive effects when combined with titanium dioxide and zinc oxide. Quercetin and rutin were prepared as 10% oil-in-water emulsions, and the resulting SPF values were 4.52 and 4.72, respectively. Therefore, they can be compared to HMS, which has a similar SPF value. In combination with titanium dioxide and quercetin, a synergistic effect and a significant increase in SPF value were observed, as well as with rutin. However, when these flavonoids were combined with zinc oxide, only an additive effect and a negligible increase in SPF value were noticed. In addition, these two flavonoids have also been shown to provide photoprotection in the UVA range.

#### 4.1.3. Apigenin

Apigenin is found in various fruits and vegetables like apples, cherries, grapes, broccoli, celery, beans, and leeks, as well as in herbs (clove, German chamomile, and endive) and beverages (wine and tea) [20]. Like other flavonoids, apigenin has shown antioxidant and photoprotective activities when applied orally and topically [81]. Apigenin has absorption peaks within UVC and UVA spectral ranges [81,82], indicating that it can only be used as a UVA filter. However, its absorbance values within the UVB range were consistently high [83]. Stevanato et al. determined in vitro the SPF value of 7% (*w*/*v*) formulation of apigenin and it was 28.8 [62]. The protective potential of apigenin against UVA and UVB-induced skin damage has been demonstrated in vitro on human keratinocytes [81].

#### 4.1.4. Luteolin

Luteolin is found in vegetables such as celery, broccoli, and carrots [84]. It possesses antioxidant [85], anti-inflammatory, and antitumor activities [84], as well as a wide UV absorption spectrum in the range of 270–390 nm [86]. Luteolin shows maximum absorption in the UVA and UVC range and another smaller peak in the UVB range [87]. Mu et al. have shown the protective effect of luteolin against UVB-induced skin damage in both in vivo and ex vivo animal models [84].

### 4.2. Resveratrol

Resveratrol is a polyphenolic compound that belongs to the stilbene class and is found in grapes, raspberries, strawberries, wine, and nuts. It exhibits anticancer, anti-inflammatory, antioxidant, and antiproliferative effects [88]. The photoprotective effects of resveratrol are well established [89,90]. Resveratrol has UV-absorbing properties with maximum absorption in the UVB range. Bhattacharya et al. have developed an emulgel formulation containing 10% resveratrol which showed in vitro an SPF value of around 9.3 and significant antioxidant activity [91]. Afaq et al. [92] have found that topical application of resveratrol (25 µmol/0.2 mL acetone per mouse) to SKH-1 hairless mice reduces the activity of lipid peroxidation, cyclooxygenase, ornithine decarboxylase (ODC), and protein expression of ODC. They point out that resveratrol is a potential photochemopreventive agent against UVB-induced damage, but that further research must be conducted in higher mammals, including humans. In addition, resveratrol protects against the harmful effects of UVA radiation by enhancing the activity of superoxide dismutase and glutathione peroxidase [93].

### 4.3. Ferulic Acid

Ferulic acid is a phenolic acid found in several plants, such as pineapple, rice, oats, coffee, peanuts, and nuts [94]. Ferulic acid exhibits numerous skin benefits, including antioxidant, anti-pigmentation, anti-aging, anti-inflammatory effects, and UV protection properties [95]. Peres et al. investigated the UV protective properties of sunscreen based on ethylhexyl triazone and bis-ethylhexyloxyphenol methoxyphenyl triazine with additional ferulic acid to obtain a multifunctional sunscreen. They found that ferulic acid increased the in vivo SPF value by 37% and the in vitro UVA-PF value by 26% [96]. Ambothi et al. indicated the photochemopreventive effects of ferulic acid against UVB-induced oxidative stress, inflammation, and angiogenesis in Swiss albino mice when ferulic acid was administered intraperitoneally and topically [97]. Jesus et al. analyzed 444 commercial sunscreens to determine the presence of antioxidants. They identified the six antioxidants with the highest frequency of use, and one of them is ferulic acid [98].

### 4.4. Curcumin

Curcumin is a yellow plant pigment obtained from the rhizome of turmeric (*Curcuma longa*, *Zingiberaceae*) [99]. The UV protective activity of curcumin was confirmed by determining the SPF value of formulations containing curcumin. Dalla et al. [100] have determined in vitro SPF values of emulsion O/W (oil in water) containing various forms and concentrations of curcumin. They found that emulsion containing 2% curcumin powder had the highest SPF value of 3.2. Further, curcumin has anti-inflammatory, antioxidant, and antiproliferative properties [101]. It has been found that curcumin influences UVA- and UVB-induced damages. Jang et al. found that curcumin inhibited UVB-induced TNF-α mRNA expression and reduced matrix metalloproteinase-1 expression in keratinocytes and fibroblasts, therefore it could be used as a potential anti-aging agent [102]. Moreover, it has been proven that curcumin significantly inhibits UVA-induced ornithine decarboxylase activity in the epidermis of CD-1 mice [99]. In addition, Tsai et al. found in vivo that the topical application of curcumin to SKH-1 hairless mice inhibits UVB-induced carcinogenesis, delaying tumor onset, multiplicity, and size [103].

### 4.5. Silymarin

Silymarin is a standardized polyphenolic extract derived from *Silybum marianum* seeds, *Asteraceae*, one of the oldest known herbal plants. It comprises 70–80% flavonolignans and a 20–30% chemically undefined fraction of polyphenolic compounds. Flavonolignan silybin is the main active component [104]. Silymarin is well known for its hepatoprotective and antioxidant properties [105], and much less so for its UV protective properties [106]. The absorption peak of silymarin is in both the UVA and UVB ranges [104]. The O/W cream based on 10% silymarin provides an in vitro SPF value close to nine, similar to OMC [107]. However, it has been to be less effective as a UVA filter. UVA protection properties (in vitro UVA-PF value) can be enhanced by combining it with zinc oxide and titanium dioxide [107]. Moreover, topically applied silymarin can prevent the development of photocarcinogenesis by preventing UVB-induced immune suppression and oxidative stress [108].

**Table 3:** Chemical structures of some natural compounds that have UV protective properties and their absorption peaks.

Name	Chemical Structure	Absorption Peaks	References
Quercetin	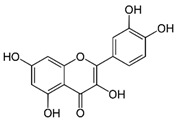	258 and 375 nm	[70]
Rutin	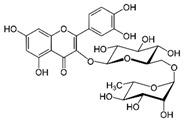	260 and 360 nm	[109]
Apigenin	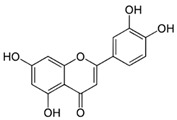	270 and 340 nm	[81]
Luteolin	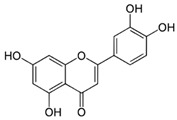	268, 284, and 333 nm	[87]
Resveratrol	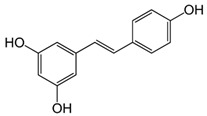	310 nm	[91]
Ferulic acid	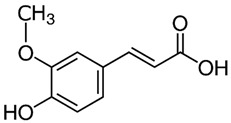	284 and 307 nm	[110]
Curcumin	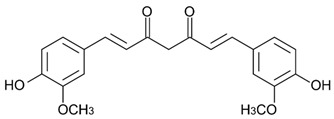	425 nm	[100]
Silymarin	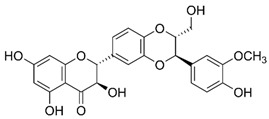	285 and 325 nm	[104]

### 4.6. Carotenoids

**Figure 3:**
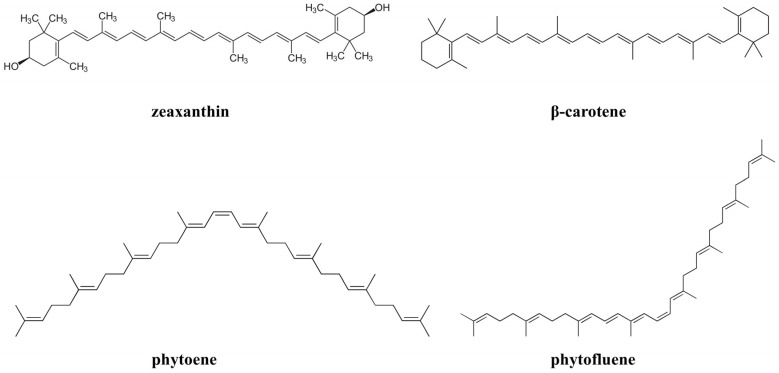
Chemical structures of some carotenoids.

Carotenoids are lipophilic pigments found in plants, animals, and microorganisms. It is known that carotenoids must be obtained through diet because the human body does not produce them. Some carotenoids such as beta-carotene, alpha-carotene, and beta-cryptoxanthin can be converted to vitamin A in the human body, so they are known as provitamins A [111]. The chemical structure of carotenoids (Figure 3) contains a system of conjugated double bonds that is responsible for their photoprotective effects: absorption of light, quenching of singlet oxygen, and scavenging free radicals [112]. Although most carotenoids have absorption peaks in the visible range, the conjugated system enables the absorption of shorter wavelengths, including UV light [113]. The chemical structure with a higher number of conjugated double bonds, like zeaxanthin and β-carotene with 11 double bonds, leads to a shift in the absorption maximum to longer wavelengths [114]. As a result, phytoene and phytofluene, which have only three and five conjugated double bonds, respectively, absorb primarily in the UV region [113] and are colorless [115]. The absorption peak of phytoene is in the UVB region (286 nm), while phytofluene is in the UVA region (348 nm) [116]. The use of carotenoids through diet and supplementation for photoprotection is well established, but topical application has also been investigated [117,118].

### 4.7. Oils

Vegetable oils have gained scientific attention for the development of sunscreen formulations because of their UV absorption properties and the improvement of the characteristics of the final products, such as absorption through the skin and spreadability [119]. Vegetable oils are composed of various compounds, thereby showing their unique chemical compositions, which determines their specific health benefits [120]. Some vegetable oils show significant SPF values reported in several studies. Kaur et al. [121] have prepared hydroalcoholic solutions of selected nonvolatile and volatile oils and investigated their sun protection potential by measuring the in vitro SPF values of formulations. Olive oil and peppermint oil showed the highest SPF values, 7.55 and 6.67, among nonvolatile and volatile oils, respectively. Nonvolatile oils are generally used as excipients in creams and lotions due to their emollient properties and production of a uniform and long-lasting film on the skin. Volatile oils are often used as perfumes in cosmetic products. Although sesame oil has an SPF value of around 2, it is a suitable candidate for the development of UV protection products due to its high antioxidant activity [122]. Aside from that, other skin benefits are its anti-inflammatory [123] and wound-healing [124] effects. Ghorbanzadeh et al. [125] have examined in vivo the potential of sesame oil in preventing UV-induced skin damage. They have found that topical application of microemulsion-based hydrogel containing 8.45 wt% sesame oil can successfully prevent UV-induced erythema, edema, skin hyperpigmentation, skin scaling, and surface roughness of the skin. Montenegro et al. have observed that formulations with organic UV filters increased the in vitro SPF values by adding 1% (*w*/*w*) pomegranate and shea oils [119].

### 4.8. Propolis

Propolis represents a plant resin collected by honeybees and its chemical composition and biological activity depend on the type of plant visited by the honeybees. The chemical composition of propolis is complex and consists of a high content of flavonoids, mainly quercetin, formononetin, pinocembrin, and coumaric acid. Propolis has UV-absorbing, anti-inflammatory, and antioxidant properties [126]. It is known that the photoprotective properties of propolis, namely its SPF value, are equal to many commercial UV filters. Almeida et al. [127] have determined the in vitro SPF values of pure lyophilized Brazilian red propolis and 1 mg/mL propolis incorporated into sunscreen formulations. The results indicated that propolis has a similar SPF value to a commercial UV filter (Filter UVA-UVB 5% Gel Permulem TR-1) and shows a synergistic effect with it. In addition, Batista et al. examined the photoprotective effect of red propolis in vivo using a rat model and found that the hydroalcoholic extract of red propolis exhibits a photoprotective effect similar to BP-3 [128]. On the other hand, Cole et al. have demonstrated that topical application of a crude ethanolic extract of Sydney propolis effectively in vivo reduces UV-induced cutaneous inflammation, immunosuppression, and lipid peroxidation [129]. Kim et al. have shown on human keratinocyte HaCaT cells that propolis significantly reduces UVA-induced ROS production and protects against UV-induced apoptosis [130].

### 4.9. Polyphenols from Waste

Bearing in mind that polyphenols are recognized as potent UV filters, affordable and sustainable resources of these compounds are very desirable. For example, extracts of grape pomace, a by-product from the winemaking industry, have been explored as sun protective agents due to their high polyphenol content. A study by Draghici-Popa et al. [131] analyzed the potential of using polyphenolic extracts of different grape marc in order to obtain sun protection creams. It was shown that 70% ethanol extract of the Merlot variety had the highest photoprotective effect with an in vitro SPF value around 7.83. This extract was later mixed with a lotion base and the obtained sun protection cream had an in vitro-estimated sun protection factor of about 10–15. Hübner et al. [132] compared in vitro photoprotective efficacy of emulsions containing only UV filters and UV filters with grape pomace extract. It was observed that formulation with the extract was 20.59% more efficient in protecting skin against UVB radiation compared with extract-free emulsion. Also, both emulsions were considered safe. Similarly, another study by the same group of authors observed synergistic behavior between grape pomace extract components and UV filters and an 81% increase in in vitro SPF value when compared with preparation containing only UV filters [133]. Therefore, grape pomace extract can be considered natural and environmentally sustainable solutions for cosmetics with UV protection properties.

Tea and coffee waste is also rich in polyphenols with high antioxidant activity that can be extracted and further used in the pharmaceutical and cosmetic industries [134,135]. The wastes resulting from olive oil production are a heterogeneous mixture of primarily antioxidant compounds, such as carbohydrates, polyphenols, and metal ions. Only 2% of phenolic compounds from olives pass into olive oil while the rest remains in waste, which makes this by-product an interesting alternative source of antioxidants for use in the pharmaceutical industry [136]. Galanakis et al. used phenolic compounds from olive mill oil wastewater to formulate different sunscreens [137]. In another study, Galanakis et al. observed that phenolics from olive mill oil wastewater provided better UV protection compared with vitamin C and vitamin E [138].

## 5. Other Natural Compounds That Contribute to UV Protection

### 5.1. Niacinamide

Niacinamide is the active, water-soluble form of vitamin B3. It possesses anti-inflammatory and antioxidant effects; therefore, it is used in the treatment of several skin disorders [98]. Moreover, topical applied niacinamide can inhibit immunosuppression caused by UVA and UVB radiation, thereby protecting the skin from photodamage [139,140]. Niacinamide is a precursor of nicotinamide adenine dinucleotide (NAD^+^) that increases ATP synthesis, consequently increasing DNA repair [141]. Due to all the above, it has been shown that nicotinamide is a promising agent for skin cancer prevention [142,143].

### 5.2. Vitamin C

Vitamin C is a powerful antioxidant and water-soluble vitamin. It is one of the most popular vitamins in topical products due to its various effects on the skin from anti-aging and anti-pigmentary to photoprotective [144]. The photoprotective effects of vitamin C include a reduction in erythema, sunburn, and immunosuppression [145]. Vitamin C acts as a free radical scavenger in aqueous compartments of the cell, which can be generated by various factors, with UV exposure being the most common source. Therefore, vitamin C protects intracellular structures from oxidative stress and can regenerate vitamin E, another potent antioxidant [146,147]. The antioxidant mechanisms of vitamin C are based on its ability to donate a hydrogen atom and form a relatively stable ascorbyl-free radical [148]. In addition, vitamin C can be used in the treatment of sunspots because it inhibits the effect of tyrosinase, an enzyme involved in the synthesis of melanin precursors [149].

### 5.3. Vitamin E

Vitamin E is a lipid-soluble vitamin that has multiple beneficial effects on the skin [150]. It is a potent antioxidant, like vitamin C, that protects cell membranes from oxidative stress by scavenging free radicals [151,152]. Vitamin E has a chromane ring with a hydroxyl group that serves as a hydrogen atom donor, enabling it to scavenge free radicals. Additionally, its hydrophobic side chain allows penetration into biological membranes [153]. In addition, when applied topically, vitamin E reduces immunosuppression, photoaging, and skin cancer [145,154]. It has been reported that vitamin E and its derivates can reduce UV-induced erythema and edema [150].

Lin et al. [155] have demonstrated in vivo that a combination of 15% vitamin C and 1% vitamin E provided higher protection against sunburn and erythema after UV exposure than either vitamin C or vitamin E alone applied topically to white Yorkshire pigs at the same concentration. Thus, vitamins C and E act synergistically to protect against UV-induced photocarcinogenesis and photoaging.

**Figure 4:**
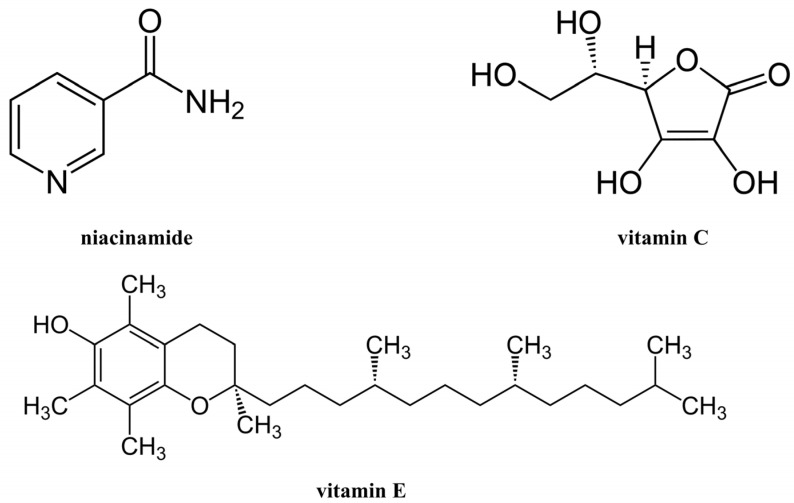
The chemical structures of some vitamins that contribute to protection against UV radiation.

Figure 4 shows the chemical structures of niacinamide, vitamin C, and vitamin E.

## 6. Limitations of Natural Compounds

Neither the FDA nor the European Union recognizes any natural compounds as approved UV filters for use. Further implementation of regulation is necessary for these compounds in order to enable the production and marketing of pharmaceutical and cosmetic products containing natural compounds as UV filters.

Some of the mentioned natural compounds as potential UV filters, such as flavonoids, have low solubility in water which can contribute to poor in vivo absorption and limited ability to penetrate through the skin [156,157]. The development of adequate formulations such as liposomes, nanostructured lipid carriers, and nano-emulsions can solve the problem of low solubility and enable the delivery of effective doses of the natural active ingredient to the epidermis [126].

One of the disadvantages of topical preparations based on vitamins C and E is the possibility of breaking the stability of such preparations when they are opened and exposed to air and light [154]. In addition, vitamin C is a powerful antioxidant, but it can also be a pro-oxidant in the presence of free transition metals [148]. Adverse effects of topically applied vitamin E such as contact dermatitis, xanthomatous reaction, and erythema are rare [154]. Resveratrol that can be found naturally in food is trans-resveratrol. However, during exposure to UV radiation, it isomerizes to a less bioactive cis form, which can be a problem in sunscreens [158].

Additionally, more studies, particularly on humans, are needed to evaluate the mechanisms of action of natural alternatives of current UV filters, as well as their safety in humans. These efforts would enable their recognition as effective and safe UV filters by the aforementioned regulatory bodies.

## 7. Conclusions

Although commercial UV filters (inorganic and organic) possess sufficient UV protective properties, their safety is a concern for users. Further research is required to identify safer and equally effective alternatives. Natural compounds can be potential alternatives to commercial UV filters, as they have shown great skin benefits due to their UV-absorbing, antioxidant, and anti-inflammatory activities. Therefore, most researchers investigate their efficacy in UV protection to replace or reduce the amount of conventional UV filters in sunscreens. Flavonoids can absorb both UVA and UVB rays and possess antioxidant, anticarcinogenic, and anti-inflammatory effects that contribute to photoprotection. Apart from flavonoids, other natural compounds such as resveratrol, ferulic acid, curcumin, silymarin, carotenoids, oils, and propolis also have UV protective properties. On the other hand, some vitamins like niacinamide, vitamin E, and vitamin C do not absorb UV rays, but they have shown effects that may contribute to UV protection. Further research is needed regarding the introduction of regulations that would enable the use of these compounds in commercial sun protection products in the future.

## Figures and Tables

**Table 1:** Sunscreen active ingredients approved by the FDA for OTC human use, their chemical structures, and their category.

Active Ingredient	Synonymous Names	Chemical Structures	FDA Category
Zinc oxide	/	ZnO	I
Titanium dioxide	/	TiO_2_	I
Para-aminobenzoic acid (PABA)	4-aminobenzoic acid	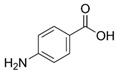	II
Trolamine salicylate	Triethanolamine salicylate	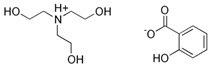	II
Avobenzone	Butyl methoxydibenzoylmethanem (BMDBM)	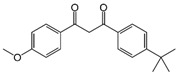	III
Cinoxate	2-Ethoxyethyl p-methoxycinnamate	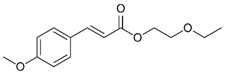	III
Dioxybenzone	Benzophenone-8 (BP-8)	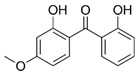	III
Ensulizole	2-phenylbenzimidazole-5-sulfonic acid	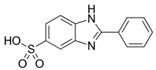	III
Homosalate (HMS)	Homomenthyl salicylate	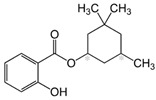	III
Meradimate	Menthyl anthranilate	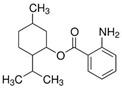	III
Octinoxate	Octyl methoxycinnamate (OMC) Ethylhexyl methoxycinnamate (EHMC)	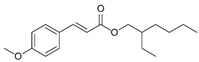	III
Octisalate (OS)	2-Ethylhexyl salicylate	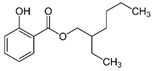	III
Octocrylene	2-Ethylhexyl 2-cyano-3,3-diphenylacrylate	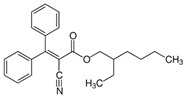	III
Oxybenzone	Benzophenone-3 (BP-3)	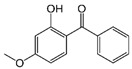	III
Padimate O	Octyl dimethyl p-aminobenzoate (OD-PABA)	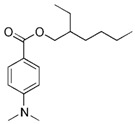	III
Sulisobenzone	Benzophenone-4 (BP-4)	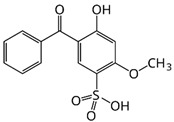	III

**Table 2:** The impact of organic UV filters on the environment.

Environmental Toxicity	UV Filter	References
detected in the seawater	Oxybenzone	[33,34]
	Octinoxate	[34]
	Octocrylene	[33,34]
	Homosalate	[33,34]
	Octisalate	[33]
detected in the coral tissues	Oxybenzone	[33,35]
	Dioxybenzone	[35]
	Octocrylene	[33,35]
	Homosalate	[33]
	Octisalate	[33]
can cause the bleaching of corals	Oxybenzone	[36]
	Dioxybenzone	[37]
	Octinoxate	[38]
	Octocrylene	[37]
toxic to some marine bacteria inducing bacterial growth inhibition	Oxybenzone	[39]
	Octinoxate	[39]
	Homosalate	[39]
found in marine bivalves	Oxybenzone	[34,40]
	Sulisobenzone	[40]
	Octinoxate	[34,40,41,42]
	Octocrylene	[34,40,41,42]
	Homosalate	[34]
	Padimate O	[42]
	Octisalate	[43]
	Enzacamene	[43]
found in fish	Oxybenzone	[44,45,46,47]
	Octinoxate	[44,45,46,47,48]
	Octocrylene	[44,45,47]
	Homosalate	[43]
	Enzacamene	[46]
found in sludge and effluent	Oxybenzone	[45,49]
	Octinoxate	[45,49]
	Octocrylene	[45,49]
	Enzacamene	[49]
found in sediment	Oxybenzone	[34]
	Octinoxate	[45]
	Octocrylene	[45]
	Homosalate	[34]

## Data Availability

Not applicable.
